# Autodidacticism and Music: Do Self-Taught Musicians Exhibit the Same Auditory Processing Advantages as Formally Trained Musicians?

**DOI:** 10.3389/fnins.2020.00752

**Published:** 2020-07-21

**Authors:** Benjamin Rich Zendel, Emily J. Alexander

**Affiliations:** ^1^Faculty of Medicine, Memorial University of Newfoundland, St. John’s, NL, Canada; ^2^Aging Research Centre – Newfoundland and Labrador, Grenfell Campus, Memorial University, Corner Brook, NL, Canada; ^3^Program in Psychology, Grenfell Campus, Memorial University, Corner Brook, NL, Canada

**Keywords:** musicianship, music training, auditory processing, MMN, ERAN, speech-in-noise, P600

## Abstract

Multiple studies have demonstrated that musicians have enhanced auditory processing abilities compared to non-musicians. In these studies, musicians are usually defined as having received some sort of formal music training. One issue with this definition is that there are many musicians who are self-taught. The goal of the current study was to determine if self-taught musicians exhibit different auditory enhancements as their formally trained counterparts. Three groups of participants were recruited: *formally trained musicians*, who received formal music training through the conservatory or private lessons; *self-taught musicians*, who learned to play music through informal methods, such as with books, videos, or by ear; *non-musicians*, who had little or no music experience. Auditory processing abilities were assessed using a speech-in-noise task, a passive pitch oddball task done while recording electrical brain activity, and a melodic tonal violation task, done both actively and passively while recording electrical brain activity. For the melodic tonal violation task, formally trained musicians were better at detecting a tonal violation compared to self-taught musicians, who were in turn better than non-musicians. The P600 evoked by a tonal violation was enhanced in formally trained musicians compared to non-musicians. The P600 evoked by an out-of-key note did not differ between formally trained and self-taught musicians, while the P600 evoked by an out-of-tune note was smaller in self-taught musicians compared to formally trained musicians. No differences were observed between the groups for the other tasks. This pattern of results suggests that music training format impacts auditory processing abilities in musical tasks; however, it is possible that these differences arose due to pre-existing factors and not due to the training itself.

## Introduction

### Musical Training and Auditory Processing

Musicians are known to have enhanced auditory processing abilities compared to non-musicians. These enhancements are associated with both functional and structural differences in the brains of musicians compared to non-musicians (Kraus and Chandrasekaran, 2010; Herholz and Zatorre, 2012). Longitudinal studies have confirmed that at least some of these advantages are due to music training and not pre-existing auditory advantages (Fujioka et al., 2006; Lappe et al., 2008, 2011; Tierney et al., 2015; Dubinsky et al., 2019; Fleming et al., 2019; Zendel et al., 2019). One commonality between both the cross-sectional and longitudinal research is that musicians and those that are given music training as part of research are trained formally. For cross-sectional studies, musicians are almost always defined as having had some sort of formal training. For longitudinal studies, non-musicians are provided a teacher or a curriculum as a form of music training. Given that music training has the potential to be used as a form of auditory rehabilitation for older adults or those with other hearing difficulties (Alain et al., 2014; Zendel and Sauvé, 2020), one important question is if formal training is necessary to enhance auditory processing abilities in musicians. It is possible that auditory enhancements observed in musicians are due to music skill acquisition, and not formal training specifically. In other words, being a musician confers auditory processing benefits, and that the training format is irrelevant. This question is important because formal training is not necessary to become a musician; many musicians are self-taught. Here we examine if formal training is critical for auditory perceptual benefits by comparing Formally Trained Musicians (FTmus), Self-Taught Musicians (STmus), and Non-Musicians (Nmus) on a series of auditory processing tasks.

While there are countless auditory processing tasks in the scientific literature, when we examine auditory processing advantages in musicians, auditory processing tasks can be roughly divided into three categories: general auditory abilities, music perception abilities, and other domain specific auditory abilities (e.g., speech perception). Importantly, the musician advantage is different in these three categories. While there are many ways to assess each of these auditory processing categories, we have chosen three commonly used behavioral and electrophysiological techniques to use in the current study: the Mismatch Negativity (MMN; general auditory abilities), the ability to detect a tonal violation in a melody, the associated Early Right Anterior Negativity (ERAN) and P600 (music perception abilities), and the ability to understand speech when there is background noise (other domain specific auditory abilities). While these measurements cover a wide variety of auditory processing tasks, there is still some disagreement if all of these abilities are enhanced in formally trained musicians. Therefore, in addition to comparing STmus to FTmus on these auditory processing tasks, we will also be able to replicate previous studies that have reported advantages in formally trained musicians on these auditory processing tasks by comparing FTmus to Nmus.

One important consideration when examining the impact of music training on auditory processing abilities is the difference between studies that compare pre-existing groups of musicians to non-musicians and, studies which provide music training to non-musicians that measure their performance before and after the training. These two types of studies are highly complementary. For longitudinal research, it is challenging to examine the development of musicianship because becoming a musician takes many years, thus most longitudinal studies focus on music-training on a shorter time scale (i.e., weeks – months). Conducting a multi-year training study is challenging, and fraught with ethical issues. For example, it would be unethical to randomly assign a participant to remain a non-musician for their entire life. Cross–sectional studies overcome these issues by allowing us to examine the impact of long-term music training, but they suffer from a lack of ability to make causal inferences associated with musical training. That is, auditory advantages in musicians could be due to music training, but could also be due to pre-existing auditory advantages. By considering cross-sectional studies on musicians in concert with longitudinal studies that provide short-term music training, we can make inferences about the life-long impacts of musical training. The current study focused on pre-existing groups of FTmus, STmus, and Nmus. Findings from this study will be directly relevant to the design and implementation of longitudinal studies that investigate if music training can be used to improve hearing abilities because the findings will provide guidance on how to design a music-training paradigm. If auditory processing advantages are similar in FTmus and STmus, then it is likely that music-based forms of auditory rehabilitation could be self-directed. On the other hand, if STmus have reduced auditory processing advantages compared to FTmus, then it is likely that having some sort of formal instruction is critical for developing empirically supported music-based forms of auditory rehabilitation. At the same time, it is critical to acknowledge that teaching oneself music in early adolescence may be fundamentally different than guiding oneself through music-based forms of auditory rehabilitation later in life. Accordingly, self-guided and teacher-guided forms of music-based auditory rehabilitation will need to be tested in an appropriate sample of participants to fully determine their potential efficacies.

### General Auditory Skills

One of the most basic auditory processes is the ability to discriminate between two tones that differ in frequency. It has been shown that formally trained musicians are able to detect smaller frequency differences in pitch compared to non-musicians (Spiegel and Watson, 1984; Kishon-Rabin et al., 2001; Micheyl et al., 2006; Parbery-Clark et al., 2009). One technique that has been used to investigate the neural mechanisms that support pitch discrimination is the oddball paradigm (Näätänen et al., 2007). In general, this paradigm involves monitoring electrical brain activity while a participant is presented with a series identical tones. Rare, deviant tones are randomly inserted into the stream of identical tones. In the case of frequency discrimination, these deviants differ from the standard tone in frequency. When a listener is ignoring the auditory environment (e.g., reading a book, watching a silent movie, or performing an unrelated task), the deviant tones evoke a mismatch negativity (MMN; Näätänen et al., 2007). The MMN is an increase in negative going electrical brain activity over fronto-central electrodes that occurs between 150 and 250 ms after the onset of the deviant tone. When listeners are asked to detect the deviant tones two additional waves are observed, an N2b and a P3 (Näätänen et al., 2007). Accordingly, the MMN has been described as an automatic process that detects change in the auditory environment, while the N2b and P3 have been described as representing conscious perception of the change in the auditory environment. One of the more interesting findings using this technique is that the musician advantage for detecting frequency differences between tones seems to be associated with the N2b/P3 and not the MMN. For example, the MMN evoked by frequency deviants without any supportive context in a passive listening paradigm (i.e., listeners were instructed to attend away from the auditory environment by reading a book or watching a silent film) was not different between formally trained musicians and non-musicians (Koelsch et al., 1999; Brattico et al., 2001; Tervaniemi et al., 2005). When listeners were asked to detect the pitch deviants, musicians exhibited an enhanced N2b and P3 response to the deviant tone, suggesting that the pitch discrimination benefit in musicians is associated with enhanced “listening” skills (Tervaniemi et al., 2005). Alternatively, it is possible that the enhanced N2b and P3 responses in musicians represent other cognitive factors associated with performing auditory tasks, including facilitated decision making for auditory judgments, or enhanced confidence in making auditory discriminations.

### Music Processing

Another interesting finding from the studies that examined differences between musicians and non-musicians on pitch discrimination tasks was when the pitch change required abstracting a musical feature from the incoming stimuli. Detecting the direction of pitch change in a series of tones, a note that violates tonal structure in a melody, or a mistuned note in a chord, all require abstracting a second-order feature (e.g., change in contour, violation of musical melodic expectancy, violation of musical harmonic expectancy). The MMN evoked by an abstract feature was first described by Saarinen et al. (1992). When the abstract feature is a violation of expectancy in music, the resulting MMN-like brain response tends to be slightly right lateralized, and has been called an Early Right Anterior Negativity (ERAN; Koelsch, 2009). The ERAN can be evoked by both mistuned chords, and by out-of-tune notes in a melody (Koelsch and Jentschke, 2010), and like the MMN, the ERAN can be evoked when attention is focused away from the auditory environment (Koelsch et al., 1999; Brattico et al., 2001). In these studies, the deviant can be contextualized simultaneously (i.e., a mistuned note in a chord; Koelsch et al., 1999), or sequentially (i.e., one note is out-of-tune in the ongoing melodic context; Brattico et al., 2001). Interestingly, the ERAN was enhanced in formally trained musicians when evoked by mistuned chords (Koelsch et al., 1999, 2002, 2007; Koelsch and Sammler, 2008; Brattico et al., 2013) but not for out-of-tune notes in a melody (Kalda and Minati, 2012). There is some evidence that ERAN-like responses to violations of melodic-like stimuli are enhanced in musicians; however, the stimuli in these studies was highly predictable (i.e., well-known melodies or repetitive patterns; Magne et al., 2006; Vuust et al., 2012). Interestingly, Kalda and Minati (2012) found that the ERAN was enhanced in musicians compared to non-musicians when evoked by a tonal deviant in a scrambled melody (i.e., highly unpredictable), but not an intact melody.

This leads to an important consideration for the ERAN: are the enhancements observed in musicians associated with attention, or there is an enhancement to the automatic encoding of tonal features in music. For musical chords, the benefit seems to occur without attention, as musicians had an enhanced ERAN when compared to non-musicians while attention was directed away from the tonal violations (Koelsch et al., 2002; Koelsch and Sammler, 2008; Brattico et al., 2013). For sequential stimuli the ERAN was enhanced in musicians compared to non-musicians when attention was directed away from the auditory environment, but only when the sequential stimuli were highly unpredictable, or highly predictable. There was no enhancement in musicians when the tonal violation was presented in a melody (Kalda and Minati, 2012). Given that only one study has compared musicians and non-musicians using melodic stimuli, interactive impact of musicianship and attention on ERAN amplitude for sequentially presented stimuli remains unclear.

Like the MMN, when listeners are asked to actively detect a mistuned chord or an out-of-tune note in a melody, the ERAN is followed by a P600. The P600 is positive going electrical wave associated with the conscious integration of the violation into the current tonal context (Besson and Faita, 1995; Janata, 1995; Patel et al., 1998; Brattico et al., 2006). Few studies have compared the impact of musicianship on P600 amplitude, and the results have been inconclusive. Besson and Faita (1995) reported an enhanced P600 response in musicians compared to non-musicians when evoked by an out-of-key note in a melodic context. At the same time, the P600 was not different in musicians and non-musicians when evoked using chords (Regnault et al., 2001; Fitzroy and Sanders, 2013). This overall pattern of results suggests that musicians are better at processing musical material. When information about a musical error occurs harmonically, the musician advantage may be due to enhanced automatic processing of the harmonic relationships, and is reflected in an enhanced ERAN. When information about a musical error occurs melodically, the musician advantage is may be associated with actively comparing and integrating the violation into the tonal context, and is reflected by an enhanced P600.

### Non-musical, Domain Specific Auditory Skills

The other main domain of auditory perception is the perception of speech, and one of the most challenging speech tests is understanding speech when there is loud background noise. Several studies suggest that musicians, compared to non-musicians, have an enhanced ability to understand speech-in-noise (Parbery-Clark et al., 2009, 2012; Zendel et al., 2015b; Slater and Kraus, 2016). At the same time, a number of other studies have failed to identify a musician advantage for understanding speech-in-noise (Ruggles et al., 2014; Boebinger et al., 2015; Madsen et al., 2017). One study found that the musician advantage for understanding speech-in-noise was moderated by age, where older musicians exhibited an advantage, but not younger musicians (Zendel and Alain, 2012). Interestingly, a recent review of 29 studies that examined the ability to understand/detect speech/signal in background noise, found that 27 of those studies reported at least one condition where musicians outperformed non-musicians (Coffey et al., 2017a). Unfortunately, the studies reviewed by Coffey et al. (2017a) did not lead to clear predictions about why musicians have an advantage at processing speech in background noise. Coffey et al. (2017a) point out that speech in noise tasks can be solved (i.e., speech is understood) through various mechanisms, and the current research in the area has yet to systematically tease apart the underlying processes involved in understanding speech in noise and the impact of musical training on each of these processes. What can be said is being a musician is likely associated with a small and variable advantage in understanding speech when there is loud background noise.

### Defining Musicians

Although there is ample evidence that being a musician is associated with enhanced auditory processing abilities, there is no good definition of what constitutes a musician. Margulis (2008) provides an excellent review of the challenges associated with defining what constitutes a musician for neuroscientific research. The main challenge is that there is a variable definition of musicianship between research groups, and in some cases within the same research group (Margulis, 2008). Most studies use two criteria for defining a musician: 1. some minimal amount of formal training, and 2. some minimal amount of daily/weekly practice. Critically, the amount of formal training and practice vary considerably between studies. Margulis (2008) points out that a musician who practices for an hour each day is fundamentally different then a musician who holds a chair in professional orchestra. Yet, in many studies both would be defined as a musician. In order to create an adequate definition of *musician* for use in neuroscientific research, it is critical to explore how individual differences in people who play musical instruments impact performance on the assessments that have been used to demonstrate that *musicians* have enhanced auditory abilities compared to *non-musicians*. The current study explored differences in training history by comparing those who received formal lessons to those that self-taught. The next sections will review some of the research exploring how individual differences in musicians lead to different outcomes on various neuro-cognitive assessments.

#### Current Status

A number of studies have examined differences between musicians based on their current musical situation (Schneider et al., 2002; Tervaniemi et al., 2006; Oechslin et al., 2013; Rogenmoser et al., 2017). Studies have compared professional to amateur musicians, concentrating on musicians with extensive, high-intensity formal music training, but differing in outcomes, where some become professionals and others continue with music as an amateur or just for fun (Schneider et al., 2002; Tervaniemi et al., 2006; Oechslin et al., 2013; Rogenmoser et al., 2017). In these studies, nearly all musicians had formal music training (Oechslin et al., 2013; Rogenmoser et al., 2017), while some studies did not specify what type of training, if any, amateur musicians had received (Schneider et al., 2002; Tervaniemi et al., 2006). Most often, the distinctions between professional and amateur musicians include hours of practice per week, level of performance, number of years of practice, and type of musician. Amateur musicians may practice less often on average than professional musicians, and have a lower training intensity (Oechslin et al., 2013). Importantly, studies have investigated similarities and differences in auditory processing between professional and amateur musicians; these studies provide support for the idea that amateur musicians have similar auditory benefits as professional musicians compared to non-musicians (Schneider et al., 2002; Tervaniemi et al., 2006; Oechslin et al., 2013; Rogenmoser et al., 2017). However, these studies also lead to questions about defining a musician based on outcomes (i.e., amateur vs. professional or hours of current practice) and not the training path which led the individual to becoming a musician.

#### Style and Instrument

A number of studies have investigated the training path by exploring differences between types of musicians. Studies have explored differences between folk, classical, jazz, and rock musicians, as well as differences between musicians who play different instruments (e.g., percussionists, pianists, violinists; Slater and Kraus, 2016). Significant differences between musicians based on the style of music or instrument they trained on are particularly relevant to the current study as they demonstrate that training format can have an impact on auditory processing abilities within musicians. Slater and Kraus (2016) tested the hypothesis that rhythmic abilities were critical for understanding speech-in-noise. They found that people with better rhythmic discrimination ability were better able to understand sentences in background noise. Supporting this relationships, they found that percussionists were better able to understand sentences in background noise compared to both vocalists, and to non-musicians (Slater and Kraus, 2016). Tervaniemi et al. (2016) found that musicians who trained in classical, jazz or rock music were most sensitive to different types of deviants using a MMN paradigm. Specifically, MMN amplitude was highest for tuning deviants in classically trained musicians; MMN amplitude was highest for timing deviants in both classically trained and jazz-trained musicians; MMN amplitude was highest for transposition deviants in jazz-trained musicians; and MMN amplitude was highest for pitch contour deviants in jazz-trained and rock-trained musicians (Tervaniemi et al., 2016). Other studies have found auditory processing specializations for musicians who play different instruments (Shahin et al., 2003; Bangert and Schlaug, 2006). Bangert and Schlaug (2006) analyzed the structure of the hand region in the motor cortex, and found that the right hemisphere region (controls left hand) was more developed in violin players, while the left hemisphere was more developed in piano players. This makes sense, considering that violin players need to make fine-grained movements with their right hand (controlled by left hemisphere), while piano players need to make fine-grained movements with both hands. Shahin et al. (2003) found that the P2 and N1c components of the auditory evoked response were enhanced in musicians compared to non-musicians. Importantly, this enhancement was larger in violin players when the evoking stimulus had the timbre of a violin, and was larger in pianists when the evoking stimulus had the timbre of a piano (Shahin et al., 2003). These comparison studies provide support for the idea that different types of training are associated with different neurophysiological outcomes.

#### Age-of-Music-Training Onset

Some of the earliest studies that examined neurophysiological differences between musicians and non-musicians found that the age at which a musician started training impacted the degree of enhancement compared to non-musicians (Schlaug et al., 1995; Amunts et al., 1997). In terms of auditory processing abilities, early trained musicians (i.e., started training before age 7) have shown enhanced abilities to replicate a rhythm compared to later trained musicians (i.e., started training after age 7; Bailey and Penhune, 2010, 2012). Interestingly, the benefit of music training on rhythmic auditory abilities increased as the age-of-music-training-onset decreased for early trained musicians only; there was no effect of age-of-music-training-onset for late-trained musicians (Bailey and Penhune, 2013). The age-of-music-training-onset has also been associated with improved abilities in understanding speech in noise (Zendel et al., 2015b; Coffey et al., 2017b). Musician participants in both of these studies included individuals who started before and after age 7 (age 2–15: Zendel et al., 2015b; age 5–12: Coffey et al., 2017b), but neither study explored this relationship beyond reporting linear correlations between age-of-training-onset and performance on a speech-in-noise task. At the same time, some studies comparing musicians and non-musicians on speech-in-noise tasks do not report age-of-music-training-onset correlations (Parbery-Clark et al., 2009, 2012; Slater and Kraus, 2016); however, musician participants in these three studies all started training before age 7. Others studies have reported no relationship age-of-music-training-onset and the ability to understand speech in noise (Boebinger et al., 2015). In many case age-of-music-training-onset is not reported and not connected to auditory processing abilities measured behaviorally or neurophysiologically (Besson and Faita, 1995; Koelsch et al., 1999, 2002, 2007; Tervaniemi et al., 2005; Koelsch and Sammler, 2008; Brattico et al., 2013). Overall, there may be a critical period for music-training-related neuroplasticity around age 7–8, where each year of training before the end of the critical period results in an additive benefit to auditory processing, while after the critical period, only years of training and current amount of practice predict performance on auditory processing tasks.

If there are differences in auditory processing abilities between formally trained musicians who learned different instruments or different styles, then it is likely that self-directed music training could also impact any auditory processing benefits associated with being a musician. This is a critical question because learning independently could be a preferred way to learn music for many. Given that learning music could be useful as a form of auditory rehabilitation (Alain et al., 2014; Zendel and Sauvé, 2020), identifying if formal training is critical to actualize auditory processing benefits is of utmost importance. We are unaware of any studies that have explicitly compared groups of musicians that are similar in their current musical status (i.e., hours of practice per week, status as a musician), but differ in terms of the training which got them there (i.e., formal training vs. self-taught).

### Current Study

Many musicians are self-taught (i.e. received no formal training) and there are many advantages to self-guided music training including accessibility, flexibility, and low cost. Modern technology has further facilitated self-teaching of music as there are countless resources for learning musical instruments available online. Accordingly, one important question is if self-taught musicians have the same auditory benefits as formally trained musicians. Previous work has shown that musicianship is associated with basic auditory abilities, musical auditory abilities, and speech-in-noise abilities. The goal of the current study was to compare auditory processing abilities in musicians with no formal music training (i.e., self-taught musicians) to formally trained musicians and non-musicians. To examine the potential auditory processing benefits in detail, participants completed three auditory processing tasks, each associated with either basic auditory abilities (automatic detection of pitch change), musical auditory abilities (ability to detect a tonal violation in a melody), and speech-in-noise abilities (performance on QuickSIN). It was expected that self-taught musicians would perform at the same level as formally trained musicians. This would provide support for future work investigating the potential benefits of self-directed music-based auditory rehabilitation.

## Materials and Methods

### Participants

Fifty-one participants were recruited through posters, online advertising and word-of-mouth. All participants provided written informed consent and the study was approved by the Grenfell Campus Research Ethics Board (GC-REB). The final sample included: 19 formally trained musicians (FTmus), 15 self-taught (STmus) musicians, and 18 non-musicians (Nmus; See Table 1 for demographic information). Formally trained musicians were defined as people who had received formal music training through either the conservatory or private lessons, with at least 5 years of formal training. Self-taught musicians were defined as people who had little to no formal music training, and who learned to play music through informal methods such as through books, online videos, tutorials, or by ear. All formally trained and self-taught musicians were actively engaged in music practice for at least 5 h per week on average in the past year. Non-musicians were defined as people who did not currently play any musical instrument, and who had little to no previous music training or experience. All participants were healthy, right-handed adults who had no neurological conditions and who were not taking psychotropic medication. All participants had a pure-tone average (i.e., average of pure-tone thresholds at 500, 1000, 2000, and 4000 Hz) below 25 dB HL in their better ear, indicating normal hearing (Humes, 2019). Participants were matched on demographic variables, except that there were more males in the STmus group compared to the other two groups, *F*(2, 51) = 3.67, *p* = 0.03, and FT musicians started their training earlier *t*(33) = 3.26, *p* = 0.003 than ST musicians.

**TABLE 1:** Participant demographics.

	**Age (years)**	**Gender**	**Non-music education (years)**	**Music practice (hours/week)**	**Music training onset (age)**	**Playing music (years)**	**Formal music training (years)**
FTmus (*N* = 19)	31.7 (*SD* = 13.7)	13 female 6 male	16.5 (*SD* = 2.7)	11.0 (*SD* = 11.1)	8.4 (*SD* = 3.3)	23.5 (*SD* = 13.8)	9.9 (*SD* = 4.3)
STmus (*N* = 15)	38.8 (*SD* = 12.9)	5 female 10 male	16.1 (*SD* = 2.4)	11.4 (*SD* = 8.4)	15.2 (*SD* = 8.2)	23.7 (*SD* = 13.0)	
Nmus (*N* = 18)	32.4 (*SD* = 13.7)	13 female 5 male	16.4 (*SD* = 2.7)				

### Stimuli and Task

All testing was carried out in a double-walled electrically shielded, sound-attenuating booth, and all stimuli were presented through Etymotic ER3A insert earphones.

#### Speech-in-Noise

After completing a demographics questionnaire and pure-tone audiometry, participants completed the QuickSIN (Quick Speech in Noise, Etymotic (Killion et al., 2004). The QuickSIN is a standardized measure of the ability to understand speech-in-noise. Participants were presented with five lists of six sentences with five key words per sentence. Each sentence was embedded in 4-talker babble noise. The sentences and noise were presented at a combined amplitudes of 70 dB SPL, using pre-recorded signal-to-noise ratios (SNRs). The SNRs decreased in 5-dB steps from 25 to 0 dB SNR. The ability to understand speech-in-noise was defined as the minimum SNR needed to identify 50% of the target words in a sentence. After each sentence was presented, participants were asked to repeat the sentence out-loud. Participants were given one point for each of the correct five words per sentence. The SNR loss was determined by subtracting the total number of words correct from 25.5, which represents the SNR required for participants to correctly identify 50% of the key words (Killion et al., 2004). To gain a stable and reliable estimate of the ability to understand speech-in-noise, scores were averaged across the five lists.

#### Active Melody Task

Next, participants were fitted with an EEG cap (BioSemi Active2). See more details below in *Recording and Analysis of Electrical Brain Activity.* A set of 40 melodies were used as stimuli for the melody task. This is the same set of melodies that was first used by Brattico et al. (2006), have since been used in a variety of music perception studies (e.g., Peretz et al., 2009; Zendel et al., 2015a; Lagrois et al., 2018; Vuvan et al., 2018). All melodies were in a major key and varied in rhythm. Melodies consisted of between 7 and 15 successive notes (*M* = 10.3, *SD* = 1.90), and were played at 120 beats per minute (500 ms per beat) at 75 dB SPL. The entire set of melodies was spread over two octaves, ranging in pitch from B4 to C5. These melodies were synthesized in six versions, varying with regard to instrumental timbre (piano or guitar) and condition (in-tune, out-of-tune, out-of-key), which resulted in 240 total melody presentations. For each melody, the pitch change was always at the same critical tone, which lasted 500 ms and was presented on the first downbeat in the third bar of the four-bar melody. Placing the target note at the same location in the melody was done for two reasons. The first was that establishing a key for the melody takes time. The second was to ensure that short-term habituation of the auditory system was similar for each target note. For In-Key melodies, all notes fell within the key of the melody. Out-of-Tune melodies contained a target note that was shifted by half a semitone from the original in-key version. This incongruity is a deviation from chromatic scale and is not a note typically used in Western music as the note would be halfway between two “legal” notes. For Out-of-Key melodies, the target note was shifted by one semitone from the in-key version. This incongruity is a deviation from the scale the melody was composed in, but is still a note in the chromatic scale. Melodies were presented in difference keys (A, B*b*, B, C, D, E*b*, F, or G) in an effort to minimize sensory novelty of tones presented in-key and out-of-key. Ten pitches were used as out-of-key targets (A, B*b*, B, C, D*b*, E*b*, E, F, G, A*b*) and nine pitches served as in-key targets (A, B*b*, B, C, D*b*, D, E, F, G*b*). There was no significant difference in the frequency of occurrence between the in-tune, out-of-tune, and out-of-key target tones,*t*(15) = 0.09, *p* = 0.93.

Participants completed three blocks of 80 trials each, during which they heard a series of melodies that sometimes contained either an out-of-tune or out-of-key note. Participants were instructed to pay attention to the melodic auditory stimuli and make a judgment about whether or not they heard a “bad” note (yes, no), and how confident they were of their response (sure, not sure). Participants were not asked to distinguish between these out-of-key and out-of-tune notes. Choices were presented on a computer screen after each melody and participants pressed a button on a response box to indicate their response.

#### Passive Melody Task

This task was identical to the active melody task, except participants were watching a silent subtitled movie, and did not have to provide a response to any of the melodies. Watching silent subtitled films does not interfere with auditory processing tasks, and allows a participant to maintain alertness without directing attention to the incoming auditory stimuli (Pettigrew et al., 2004).

#### Passive Oddball Task

Participants were presented with a stream of tones while continuing to watch a silent subtitled movie. The standard tone was a C6 piano tone (1047 Hz). The tone was 100 ms long, had rise and fall times of 10 ms and was presented at 75 dB SPL. Four oddball tones were also presented. They were identical to the standard tone except they were either shifted up or down in pitch either by 200 cents (933 or 1175 Hz) or by 25 cents (1032 or 1062 Hz). The sequence contained 900 standard tones and 200 oddball tones (50 for each oddball type). In total, 1100 tones were presented with an interstimulus interval that randomly varied between 800 and 1100 ms. The presentation of sounds was pseudo-randomized such that each oddball tone was preceded by at least four standard tones.

### Recording and Analysis of Electrical Brain Activity

Neuroelectric brain activity was collected continuously from 70 scalp locations using a high-pass filter set at 0.1 Hz, a sampling rate of 1024 Hz per channel and stored for offline analysis. Four additional electrodes, were attached around the eyes to monitor ocular activity (IO1, IO2, LO1, LO2) and two more were attached to the mastoid bones (M1, M2) to serve as a reference for data analysis.

Sets of ocular movements were obtained for each participant prior to the experiment (Picton et al., 2000). Prototypical eye blinks, lateral eye movements, and vertical eye movements were identified in the continuous EEG data. A principal component analysis (PCA) was calculated on the EEG data associated with these eye movements which created a set of movement components that best explained the eye movements. The scalp activity associated with these eye movements was subtracted from each ERP to minimize interference from ocular activity for each participant average. ERPs were then low-pass filtered to attenuate frequencies between 0.1 and 30 Hz. After this correction, trials containing excess noise (±125 μV), excluding electrodes adjacent to the eyes (i.e., IO1, IO2, LO1, LO2), were rejected before averaging.

## Results

### Behavioral Data

#### Speech-in-Noise

**FIGURE 1:**
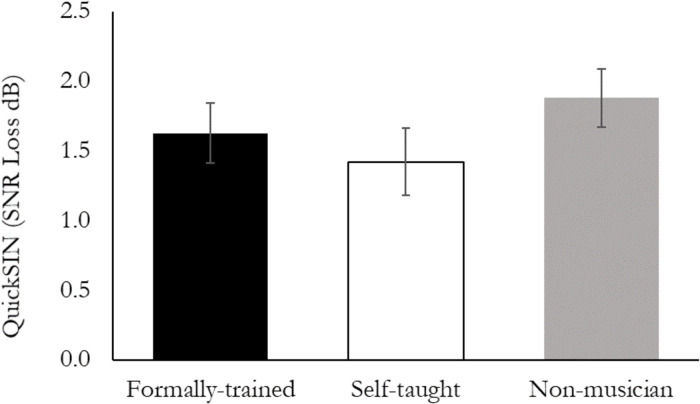
Performance on the QuickSIN task. There were no significant differences between Formally trained musicians, Self-taught musicians, or Non-musicians.

Performance on the QuickSIN test was quantified using a one-way ANOVA that included Group (FTmus, STmus, Nmus) as a between-subject factor. There was no difference between the three groups on the QuickSIN, *F*(2, 49) = 0.76, *p* = 0.47 (Figure 1).

#### Active Melody Task: Accuracy

**FIGURE 2:**
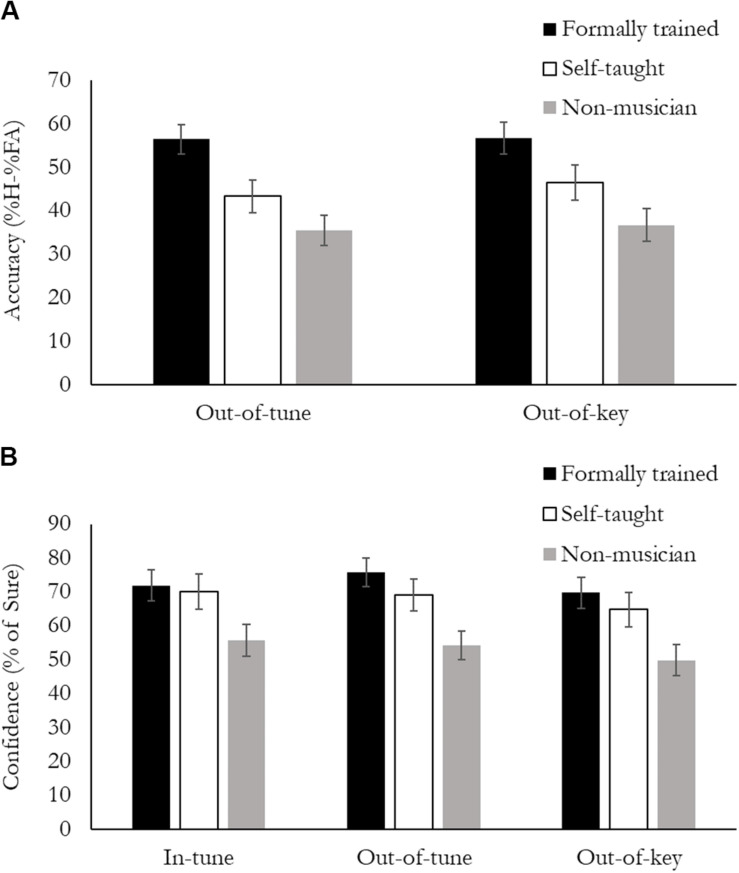
Behavioral performance during the Active Melodic Tonal Violation Task. **(A)** Accuracy was calculated as %Hits minus %False Alarms, and is presented separately for the Out-of-tune and Out-of-key violation. Formally trained musicians had higher accuracy compared to Self-taught musicians who had higher accuracy than Non-musicians for both types of violation. **(B)** Confidence was calculated as the % of trials in which the participants reported “sure” and is presented separately for the three stimuli types. Both groups of musicians were more confident than non-musicians for all stimuli.

Accuracy was calculated as a percentage of hits minus false alarms (HFA%). A hit was when a participant correctly identified that they heard a tonal violation in the melody, and a false alarm was when a participant reported hearing a tonal violation in the melody, when all the notes where In-key. Data was analyzed using a 2 (Note type: Out-of-key, Out-of-tune) × 3 (Group: FTmus, STmus, Nmus) mixed design ANOVA. Overall, there was a main effect of Group, *F*(2, 49) = 9.31, *p* < 0.001, η*_*p*_*^2^ = 0.28 (Figure 2A). FTmus had greater accuracy than both STmus (*p* = 0.02) and Nmus (*p* < 0.001), and STmus had greater accuracy than the Nmus, but this effect was not significant (*p* = 0.09). There was no difference in Accuracy between Out-of-key and Out-of-tune notes (*p* = 0.65), and the Group by Note type interaction was not significant (*p* = 0.23).

#### Active Melody Task: Confidence

**FIGURE 3:**
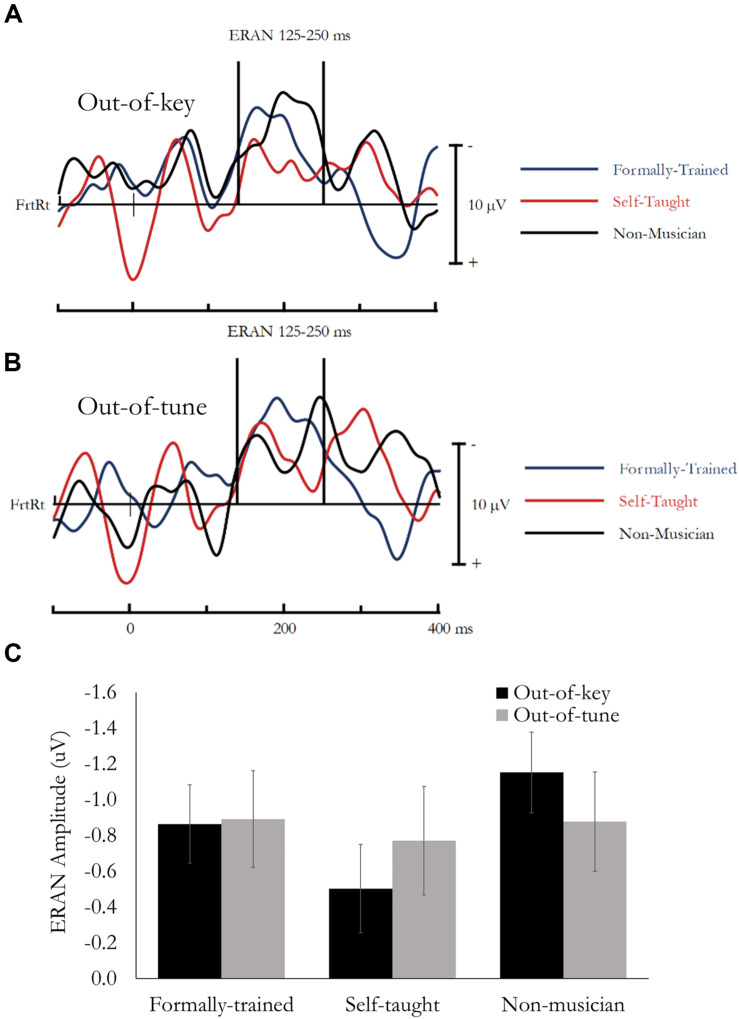
ERAN evoked during the Active Melodic Tonal Violation Task. No group differences were found for ERAN amplitude. **(A)** Difference waves (In-key minus Out-of-key) are presented separately for each group averaged across the analysis montage. **(B)** Difference waves (In-key minus Out-of-tune) are presented separately for each group averaged across the analysis montage. **(C)** Mean ERAN amplitude (125–250 ms) as a function of Group and Tonal Violation.

Confidence was calculated as the percentage of trials where the participant was “sure” of their response, regardless of their accuracy. Data was analyzed using a 3 (Note type: In-key, Out-of-key, Out-of-tune) × 3 (Group: FTmus, STmus, Nmus) mixed design ANOVA. Results can be seen in Figure 3. Overall, there was a main effect of Group, *F*(2, 49) = 5.73, *p* < 0.006, η*_*p*_*^2^ = 0.18 (Figure 2B). Nmus were less confident than both FTmus (*p* = 0.002) and STmus (*p* = 0.022). There was no difference in confidence between FTmus and STmus (*p* = 0.48). There was also a main effect of Note type, *F*(2, 98) = 5.28, *p* = 0.007, η*_*p*_*^2^ = 0.10. Participants were less confident when the Note type was Out-of-key compared to both Out-of-tune (*p* < 0.001), and In-key (*p* = 0.02). The Note type by Group interaction was not significant (*p* = 0.68).

### Electrophysiological Data

#### Active Melody Task: ERAN

The early right anterior negativity (ERAN) was quantified as the difference in amplitude between the event-related potential (ERP) evoked by the In-key and Out-of-key or Out-of-tune stimuli. Based on previous research (Brattico et al., 2006; Lagrois et al., 2018) and visual inspection of the data, the ERAN was extracted as the mean amplitude between 125 and 250 ms, from a montage of 11 fronto-right Electrodes (AFz, AF4, AF8, Fz, F2, F4, F6, FCz, FC2, FC4, FC6). Data was analyzed using a mixed design ANOVA, that included Note type (In-key, Out-of-key/Out-of-tune) and Electrode as within-subject factors, and Group as a between-subject factor. Main effects and interactions involving electrode are not reported because multiple electrodes were used to ensure a stable and reliable estimate of the ERAN. Difference waves, presenting the ERAN averaged across the 11-electrode montage, are presented in Figure 3A (In-key minus Out-of-key) and Figure 3B (In-key minus Out-of-tune). Mean amplitude for the ERAN is presented in Figure 3C.

##### ERAN: Out-of-Key

Overall, mean amplitude between 125 and 250 ms was more negative for Out-of-key notes compared to In-key notes, *F*(1, 49) = 39.77, *p* < 0.001, η*_*p*_*^2^ = 0.45. This effect reflects the ERAN, and most critically, was not different between the three groups and the Note type by Group interaction was not significant, *F*(1, 49) = 1.89, *p* = 0.16, η*_*p*_*^2^ = 0.07.

##### ERAN: Out-of-Tune

Overall, mean amplitude between 125 and 250 ms was more negative for Out-of-tune notes compared to In-key notes, *F*(1, 49) = 26.66, *p* < 0.001, η*_*p*_*^2^ = 0.35. This effect reflects the ERAN, and most critically, was not different between the three groups and the Note type by Group interaction was not significant, *F*(1, 49) = 0.05, *p* = 0.95, η*_*p*_*^2^ < 0.01.

### Active Melody Task: P600

**FIGURE 4:**
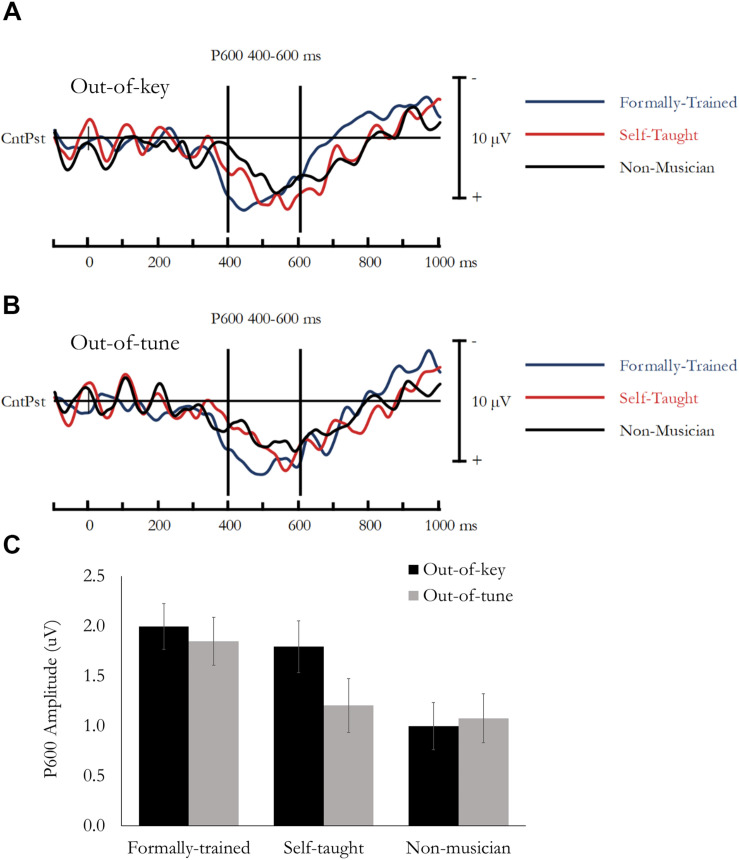
P600 evoked during the Active Melodic Tonal Violation Task. The P600 was larger in Formally trained musicians compared to the other groups when evoked by an Out-of-tune note, and the P600 was larger in both Formally trained and Self-taught musicians compared to Non-musicians when evoked by an Out-of-key note. **(A)** Difference waves (In-key minus Out-of-key) are presented separately for each group averaged across the analysis montage. **(B)** Difference waves (In-key minus Out-of-tune) are presented separately for each group averaged across the analysis montage. **(C)** Mean P600 amplitude (400–600 ms) as a function of Group and Tonal Violation.

The P600 was quantified as the difference in amplitude between the ERP evoked by the In-key and Out-of-key or Out-of-tune stimuli. Based on previous research (Brattico et al., 2006; Lagrois et al., 2018) and visual inspection of the data, the P600 was extracted as the mean amplitude between 400 and 600 ms, from a montage of nine central-parietal Electrodes (CP1, CPz, CP2, P1, Pz, P2, PO3, POz, PO4). Data was analyzed using a mixed design ANOVA, that included Note type (In-key, Out-of-key/Out-of-tune) and Electrode as within-subject factors, and Group as a between-subject factor. Main effects and interactions involving electrode are not reported because multiple electrodes were used to ensure a stable and reliable estimate of the P600. Difference waves, presenting the P600 averaged across the 9-electrode montage are presented in Figure 4A (In-key minus Out-of-key) and Figure 4B (In-key minus Out-of-tune). Mean amplitude for the P600 is presented in Figure 4C.

#### P600: Out-of-Key

Overall, mean amplitude between 400 and 600 ms was more positive for Out-of-key notes compared to In-key notes, *F*(1, 49) = 130.18, *p* < 0.001, η*_*p*_*^2^ = 0.73. This effect reflects the P600. Importantly, this effect differed between groups as the Note type by Group interaction was significant, *F*(1, 49) = 5.01, *p* = 0.01, η*_*p*_*^2^ = 0.17. Follow-up tests were done using the mean amplitude of the difference waves (i.e., Out-of-key minus In-key) averaged across the 9-electrode montage. This revealed that the P600 was smaller in Nmus compared to both FTmus (*p* = 0.04) and STmus (*p* = 0.027). There was no difference between FTmus and STmus (*p* = 0.57).

#### P600: Out-of-Tune

Overall, mean amplitude between 400 and 600 ms was more positive for Out-of-tune notes compared to In-key notes, *F*(1, 49) = 89.75, *p* < 0.001, η*_*p*_*^2^ = 0.65. This effect reflects the P600. Importantly, this effect differed between groups as the Note type by Group interaction was nearly significant, *F*(1, 49) = 2.88, *p* = 0.066, η*_*p*_*^2^ = 0.11. Follow-up tests were done using the mean amplitude of the difference waves (i.e., Out-of-tune minus In-key) averaged across the 9-electrode montage. This revealed that the P600 was larger in FTmus compared to both Nmus (*p* = 0.029) and STmus (*p* = 0.08). There was no difference between Nmus and STmus (*p* = 0.73).

#### Passive Melody Task: ERAN

**FIGURE 5:**
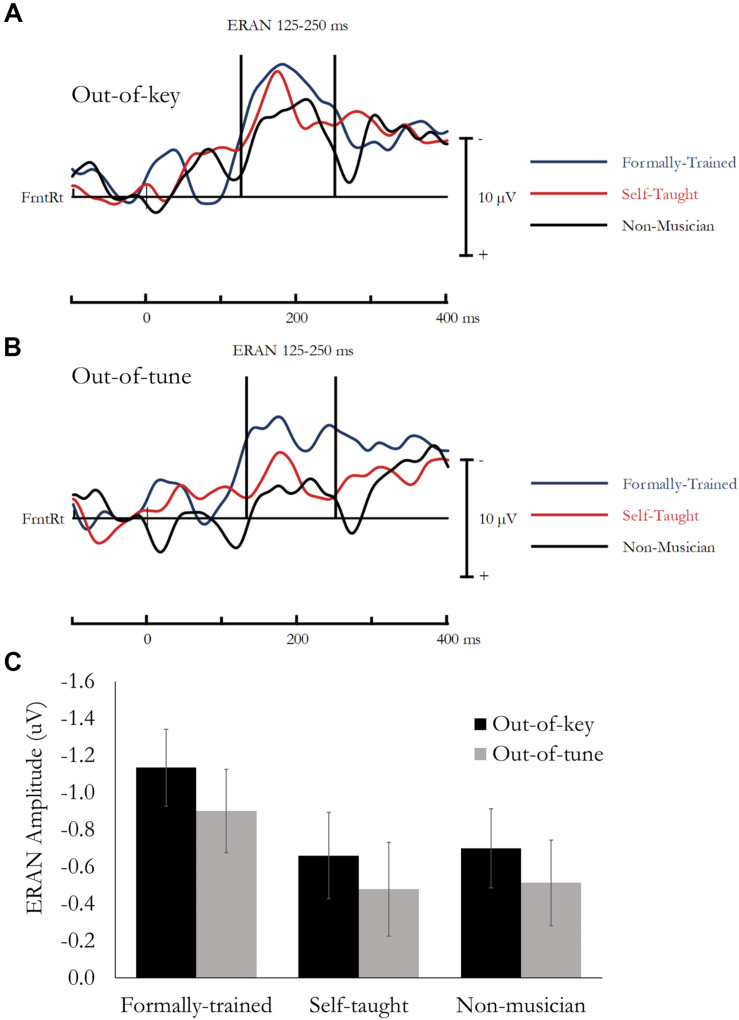
ERAN evoked during the Passive Melodic Tonal Violation Task. No group differences were found for ERAN amplitude. **(A)** Difference waves (In-key minus Out-of-key) are presented separately for each group averaged across the analysis montage. **(B)** Difference waves (In-key minus Out-of-tune) are presented separately for each group averaged across the analysis montage. **(C)** Mean ERAN amplitude (125–250 ms) as a function of Group and Tonal Violation.

The ERAN evoked during passive listening was analyzed in the same manner as the ERAN evoked during the active listening task (see above). Difference waves, presenting the ERAN averaged across the 11-electrode montage, are presented in Figure 5A (In-key minus Out-of-key) and Figure 5B (In-key minus Out-of-tune). Mean amplitude for the ERAN is presented in Figure 5C.

##### ERAN: Out-of-Key

Overall, mean amplitude between 125 and 250 ms was more negative for Out-of-key notes compared to In-key notes, *F*(1, 49) = 43.417, *p* < 0.001, η*_*p*_*^2^ = 0.47. This effect reflects the ERAN, and most critically, was not different between the three groups and the Note type by Group interaction was not significant, *F*(1, 49) = 1.52, *p* = 0.23, η*_*p*_*^2^ = 0.06.

##### ERAN: Out-of-Tune

Overall, mean amplitude between 125 and 250 ms was more negative for Out-of-tune notes compared to In-key notes, *F*(1, 49) = 21.35, *p* < 0.001, η*_*p*_*^2^ = 0.30. This effect reflects the ERAN, and most critically, was not different between the three groups and the Note type by Group interaction was not significant, *F*(1, 49) = 1.02, *p* = 0.37, η*_*p*_*^2^ = 0.04.

#### Passive Oddball Task: MMN

**FIGURE 6:**
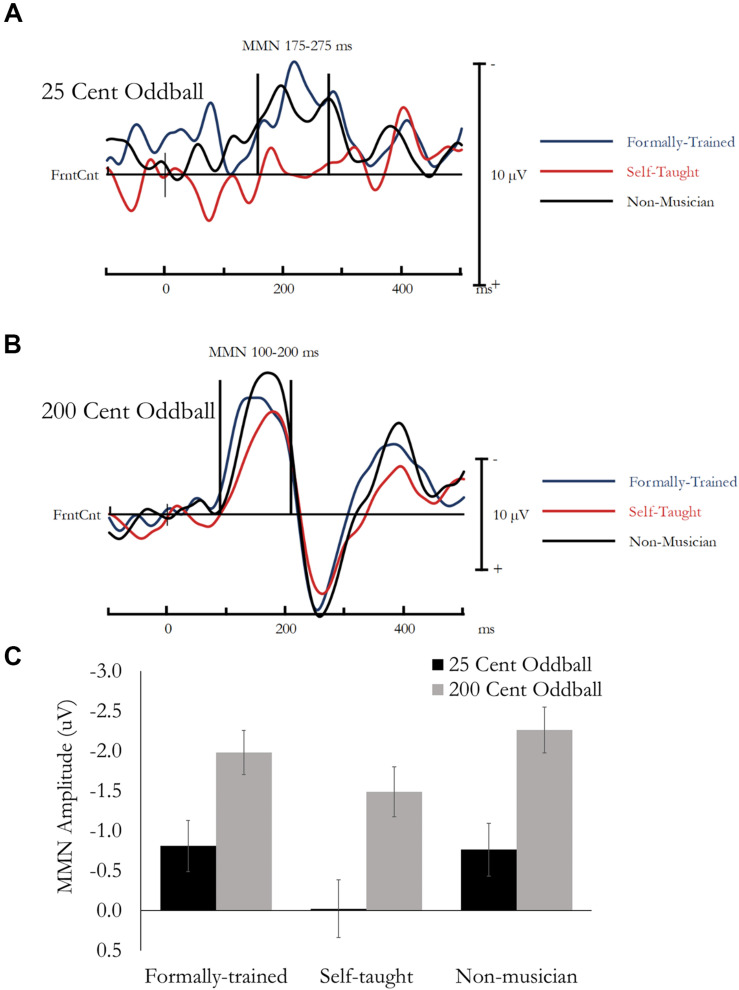
MMN evoked during the Passive Oddball Task. No group differences were found for MMN amplitude. **(A)** Difference waves (Standard minus 25 cent Oddball) are presented separately for each group averaged across the analysis montage. **(B)** Difference waves (Standard minus 200 cent Oddball) are presented separately for each group averaged across the analysis montage. **(C)** Mean MMN amplitude evoked by a 25 cent Oddball (175–275 ms) as a function of Group, and MMN amplitude evoked by a 200 cent Oddball (100–200 ms) as a function of Group.

The Mismatch Negativity (MMN) was quantified as the difference in amplitude between the ERP evoked by the Standard and the 25 cent oddball or the 200 cent oddball. Based on previous research (Näätänen et al., 2007) and visual inspection of the data, the MMN was extracted as the mean amplitude between 175 and 275 ms for the 25 cent deviant, and 100–200 ms for the 200 cent deviant, from a montage of nine fronto-central Electrodes (F1, Fz, F2, FC1, FCz, FC2, C1, Cz, C2). Data was analyzed using a mixed design ANOVA, that included Tone type [Standard, Deviant (25 or 200 cent)] and Electrode as within-subject factors, and Group as a between-subject factor. Main effects and interactions involving electrode are not reported because multiple electrodes were used to ensure a stable and reliable estimate of the MMN. Difference waves, presenting the MMN averaged across the 9-electrode montage are presented in Figure 6A (Standard minus 25 Cent Oddball) and Figure 6B (Standard minus 200 Cent Oddball). Mean amplitude for the MMN is presented in Figure 6C.

##### MMN: 25 Cent Deviant

Overall, mean amplitude between 175 and 275 ms was more negative for 25 cent Deviant tones compared to Standard notes, *F*(1, 49) = 7.45, *p* = 0.009, η*_*p*_*^2^ = 0.13. This effect reflects the MMN, and most critically, was not different between the three groups and the Tone type by Group interaction was not significant, *F*(1, 49) = 1.59, *p* = 0.22, η*_*p*_*^2^ = 0.06.

##### MMN: 200 Cent Deviant

Overall, mean amplitude between 100 and 200 ms was more negative for 200 cent Deviant tones compared to Standard tones, *F*(1, 49) = 127.56, *p* < 0.001, η*_*p*_*^2^ = 0.72. This effect reflects the MMN, and most critically, was not different between the three groups and the Tone type by Group interaction was not significant, *F*(1, 49) = 1.67, *p* = 0.20, η*_*p*_*^2^ = 0.06.

### Brain Behavior Correlations

**FIGURE 7:**
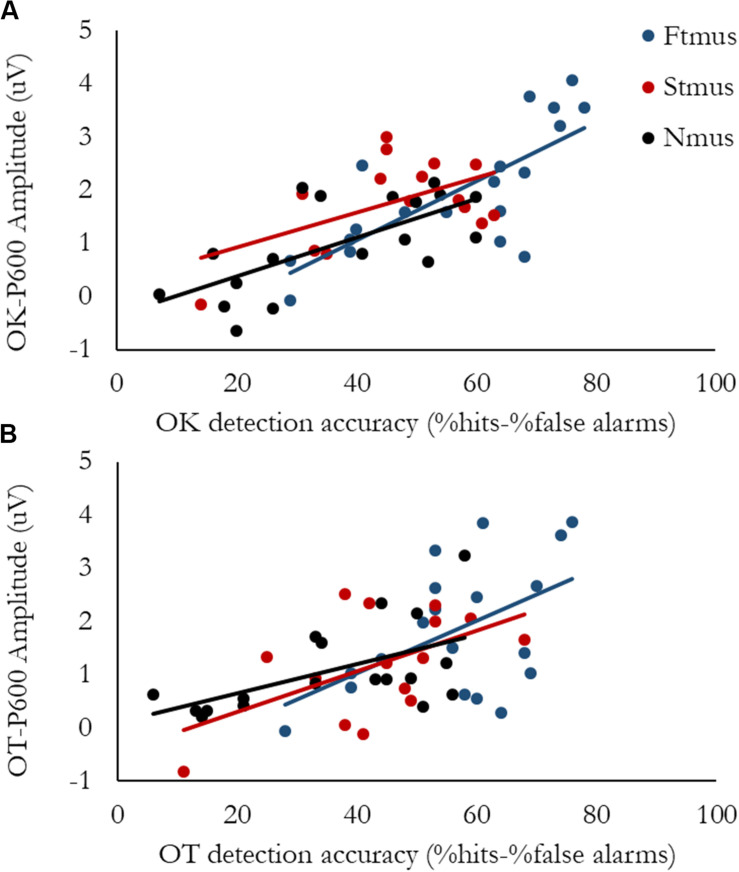
Brain-Behavior Scatterplots. Data points are separated by group, with Formally trained musicians in blue, Self-taught musicians in red, and Non-musicians in black. **(A)** P600 amplitude evoked by an Out-of-key deviant as a function of Accuracy for detecting an Out-of-key deviant. **(B)** P600 amplitude evoked by an Out-of-tune deviant as a function of Accuracy for detecting an Out-of-tune deviant.

Group differences were observed for Accuracy, therefore to explore the connection between Accuracy and neural activity, two regressions were calculated. Both examined if the passive ERAN, active ERAN, or P600 predicted Accuracy; one regression was calculated for the Out-of-key violation, and the other was calculated for the Out-of-tune violation. For the Out-of-key violation, the overall regression model was significant, *F*(3, 48) = 18.71, *p* < 0.001, *R*^2^ = 0.54. Only P600 amplitude was significantly associated with Accuracy, *t* = 6.79, *p* < 0.001. The amplitude of the ERAN recorded both during passive and active listening tasks were not associated with Accuracy, *t* = −0.77, *p* = 0.44 and *t* = 0.19, *p* = 0.85, respectively. For the Out-of-tune violation, the overall regression model was significant, *F*(3, 48) = 8.91, *p* < 0.001, *R*^2^ = 0.36. Only P600 amplitude was significantly associated with Accuracy, *t* = 4.85, *p* < 0.001. The amplitude of the ERAN recorded both during passive and active listening tasks were not associated with Accuracy, *t* = −0.95, *p* = 0.35 and *t* = −0.81, *p* = 0.42, respectively. To explore potential group differences in the relationship between P600 amplitude and Accuracy, bivariate correlations between P600 amplitude and Accuracy were calculated separately for each group. For FTmus the correlation between P600 amplitude and Accuracy was significant for both Out-of-Tune and Out-of-Key notes, r = 0.51 and 0.75, *p* = 0.03 and *p* < 0.001, respectively. For STmus the correlation between P600 amplitude and Accuracy was significant for both Out-of-Tune and Out-of-Key notes, r = 0.54 and 0.54, *p* = 0.04 and 0.04, respectively. For Nmus the correlation between P600 amplitude and Accuracy was significant for both Out-of-Tune and Out-of-Key notes, r = 0.55 and 0.68, *p* = 0.02 and 0.002, respectively. Scatterplots presenting the relationship between Accuracy and P600 amplitude are presented in Figure 7.

### Age-of-Music-Training Onset

There were significant differences in the age-of-music-training onset for the STmus and FTmus groups. In order to determine if the age-of-music-training onset had an impact on performance, a linear regression was calculated that included age-of-music-training onset as a dependent factor, and measurements where there were significant group differences (reported above) as predictors [i.e., P600 (Out-of-key and Out-of-tune), Accuracy (Out-of-key and Out-of-Tune), Confidence (Out-of-key, Out-of-tune and In-key)]. The overall regression was not significant, *F*(7, 26) = 1.74, *p* = 0.14. suggesting that age-of-music-training-onset did not impact performance on any tasks where musicians had an advantage over non-musicians in the current study.

## Discussion

This study explored auditory processing differences between formally trained musicians (FTmus), self-taught musicians (STmus), and non-musicians (Nmus) across three tasks: a speech-in-noise task, a passive pitch deviant task, and a melodic tonal violation task. Differences between the three groups were only observed for the melodic tonal violation task. FTmus were more accurate at detecting tonal violations in a melody compared to STmus, and STmus were more accurate at detecting tonal violations in a melody compared to Nmus. This enhanced ability was related to the P600 response. The P600 was enhanced in FTmus and STmus compared to Nmus when evoked by an Out-of-Key note, and was enhanced in FTmus compared to both STmus and Nmus when evoked by an Out-of-Tune note. Importantly, the differences between STmus and FTmus were not due to education levels, current hours per week of music practice, or years of music experience as the groups were matched on these variables. Differences between FTmus and STmus for the age-of-music-training-onset did not likely contribute to the group differences, as age-of-music-training-onset was not associated with musician advantages for the dependent measurements in the study where musicians had an advantage over non-musicians. No group differences were observed for MMN evoked by frequency deviants, nor for the ability to understand speech in background noise as measured by performance on the QuickSIN. It is important to acknowledge that group differences in this study could be due to neuroplasticity associated with music training, pre-existing differences between the groups that impacted an individual’s likelihood to become a FTmus, STmus, or Nmus, or a combination of both. The results show associations between task performance and music training type, not the causal impact of music training. The next section will examine the ERAN, P600, Accuracy and Confidence measurements recorded during the melodic tonal violation task. This will be followed by a discussion of the null results observed for the MMN and QuickSIN.

### Melodic Tonal Violation Task

#### Accuracy and Confidence

As was expected, FTmus compared to Nmus had higher accuracy for detecting Out-of-Key and Out-of-Tune notes. This finding replicates previous work which found that formally trained musicians have enhanced abilities to detect a tonal violation contained in a melody (Besson and Faita, 1995), and extends these findings by demonstrating that formally trained musicians compared to non-musicians are better able to detect both diatonic and chromatic violations (i.e., Out-of-key and Out-of-tune). Interestingly, the STmus were better than the Nmus at detecting both Out-of-key and Out-of-tune notes, but were not as good as the FTmus. At the same time, the STmus were just as confident as the FTmus in their ability to do this task. This pattern of results suggests that STmus are less aware of their performance compared to FTmus, as the relationship between accuracy and confidence has been shown to reflect awareness (Zendel et al., 2015a; Vuvan et al., 2018). This reduced awareness could be due to “over confidence” by the STmus, because without formal training STmus were never corrected by a teacher when they made mistakes, and therefore may tend to think they are better at detecting tonal violations then they actually are. An alternative possibility is that formally trained musicians are “under-confident” in their ability to detect a tonal violation. This could be because formal training involves constant identification of errors by a teacher. It is possible that this process could humble a formally trained musician, and make them less confident in their own musical abilities due to the hyper-awareness developed by formal training. The current study does not allow us to determine if one or both of these hypotheses is correct, but future work should examine the relationship between confidence and performance in musicians with different types of training.

#### Group Differences on P600

In the current study, Accuracy at detecting a tonal violation was predicted by the amplitude of the P600, and the P600 was enhanced in FTmus compared to Nmus. The enhanced P600 in FTmus compared to Nmus replicates previous work examining P600 responses evoked by melodic incongruities (Besson and Faita, 1995), and extends these findings by demonstrating that the P600 is enhanced in FTmus compared to Nmus when evoked by both diatonic and chromatic violations (i.e., Out-of-key and Out-of-tune notes). The main purpose of the current study was to examine how STmus performed compared to both FTmus and Nmus. Here, the type of violation was critical. For the Out-of-Key violation, the P600 in STmus was comparable to FTmus, while for the Out-of-Tune violation, the P600 in STmus was comparable to Nmus. The interpretation of the P600 results should be considered along with the behavioral data to make sense of the difference between FTmus and STmus.

When the behavioral results are considered in concert with the P600 data, an interesting pattern emerges. As was expected, FTmus always had the highest accuracy, and the largest P600, while Nmus always had the lowest accuracy, and the smallest P600. For the Out-of-Key violation, accuracy was higher in FTmus compared to STmus, but the P600 response was comparable. Detecting an Out-of-Key violation requires knowledge of diatonic musical scales. It is likely that neural responses to a diatonic violation were not different between STmus and FTmus because of their increased experience using diatonic scales during music performance or practice compared to non-musicians. Interestingly, the strength of the correlation between P600 and Accuracy for an out-of-key note was weaker in STmus compared to FTmus (0.54 vs. 0.75), suggesting that STmus are less able to utilize this enhanced neural information as their detection accuracy was lower than the FTmus. The pattern for Out-of-tune notes was different. Out-of-Tune notes are more salient than Out-of-Key notes because they violate the general chromatic scale used in all Western music, and not a diatonic scale that is specific to a melody or song. Supporting this hypothesis, previous work has shown that the ability to detect Out-of-tune notes was associated with the ERAN and not the P600, and that performance on a click-detection task was reduced when the click followed an Out-of-Tune note, but not an Out-of-Key or In-Key note (Lagrois et al., 2018). Although we did not replicate the previously reported relationship between ERAN and Accuracy; Accuracy was lower in Nmus compared to STmus, but the P600 responses in these groups was comparable. This pattern of results suggests that some additional brain process is contributing to the ability to detect tonal violations of the chromatic scale in STmus that is not associated with P600 amplitude because the correlation strength between P600 and Accuracy for detecting an Out-of-Tune note was similar in Nmus and STmus (0.55 and 0.54). The overall asymmetry between training type, tonal violation, accuracy, and P600 amplitude should be further explored to better understand the relationship between different forms of music training and the specificity of the putative training-related plasticity.

#### ERAN

An ERAN was evoked in both the active and passive versions of the melodic tonal violation task, and the ERAN did not differ between groups. This finding is important for two reasons. First, it provides more evidence that the ERAN evoked by melodic incongruities is not enhanced in musicians (Kalda and Minati, 2012). Some studies have found an enhanced ERAN evoked by single note violations in musicians; however, both these studies used stimuli that could be easily predicted by the listener [i.e., a repetitive Alberti bass pattern (Vuust et al., 2012), or familiar melodies (Magne et al., 2006)] or were entirely unpredictable (i.e., scrambled melodies; Kalda and Minati, 2012). A longitudinal study where non-musicians were given music training over the course of 2 weeks found that the ERAN evoked by a tonal violation at the end of an arpeggio of a major chord (i.e., easily predictable) was enhanced in the group that received training compared to a control group (Lappe et al., 2008). At the same time, a number of studies have observed enhanced ERAN in musicians compared to non-musicians when evoked by chords (Koelsch et al., 1999, 2002, 2007; Koelsch and Sammler, 2008; Brattico et al., 2013). It is therefore likely that the musician advantage for processing tonal information is dependent on the stimulus. The ERAN is likely enhanced in musicians compared to non-musicians when the tonal violation can be determined based on the deviant stimulus alone, without reference to preceding acoustic information (i.e., chords), or, when the preceding context makes the target stimulus easily predictable or unpredictable by the listener. This would explain the enhanced ERAN in musicians when evoked by chords, deviants in repetitive patterns, or deviants in familiar melodies. When the violation requires greater integration of preceding acoustic information, and that information moderately unpredictable, as is the case with novel melodies, the ERAN is not enhanced in musicians. Integration over time may require conscious access to the ongoing representation of tonal structure, and this would be indexed by the P600 (Besson and Faita, 1995; Janata, 1995; Patel et al., 1998; Brattico et al., 2006). Support for this proposal comes from the observation that the P600 and not the ERAN was enhanced in FTmus in the current and previous studies that used single note melodies (Besson and Faita, 1995). At the same time, the ERAN and not the P600 was enhanced in previous work when the stimuli were chords (Regnault et al., 2001; Koelsch et al., 2002, 2007; Koelsch and Sammler, 2008; Brattico et al., 2013; Fitzroy and Sanders, 2013).

### Passive Pitch Deviant Task

#### MMN

No group differences in MMN amplitude were observed in the current study. This finding replicates previous work examining the MMN evoked by small pitch deviants comparing musicians and non-musicians (Koelsch et al., 1999; Brattico et al., 2001; Tervaniemi et al., 2005). Here, we extend these findings by demonstrating that there is no difference between both self-taught and formally trained musicians. It is therefore likely that automatic detection of pitch deviants outside of a musical context is not impacted by any form of musical training. Previous work using similar paradigms has shown that when musicians are asked to actively detect the pitch deviants, that the N2b and P3 are enhanced in formally trained musicians compared to non-musicians (Tervaniemi et al., 2005). Due to time constraints, an active task was not possible in the current study, but when the MMN results are considered along with the P600 results reported above, it is likely that self-taught musicians would exhibit an increased amplitude in both N2b and P3 responses to small pitch deviants compared to non-musicians. One important question is if these putative enhancements are different than those observed in formally trained musicians. It would also be interesting to explore other auditory processing tasks in formally trained and self-taught musicians. For example, Tervaniemi et al. (2006) reported an enhanced MMN in musicians compared to non-musicians for intensity and location deviants, but not for pitch deviants. It is therefore likely that musicianship is associated with enhanced processing of multiple acoustic features, but that the advantage in processing each feature occurs at a unique processing stage. An important question that follows from the current study is if this complex pattern is similar or different in self-taught and formally trained musicians.

### Speech-in-Noise Task

There has been considerable debate about whether or not musicians have an advanced ability to understand speech-in-noise compared to non-musicians. While many studies have shown an advantage for musicians compared to non-musicians (Parbery-Clark et al., 2009, 2012; Zendel et al., 2015b; Slater and Kraus, 2016), in many others the differences have not reached statistical significance (Ruggles et al., 2014; Boebinger et al., 2015; Madsen et al., 2017). Overall, it is likely that formally trained musicians tend to understand speech in noise better than non-musicians, but these differences don’t always reach statistical significance. This is particularly evident with the QuickSIN assessment used in the current study. For example, Ruggles et al. (2014) reported that the QuickSIN scores of musicians was lower (i.e., better) than non-musicians (0.80 vs. 1.12 dB SNR loss), but that the difference did not reach statistical significance. Parbery-Clark et al. (2009) and Slater and Kraus (2016) both reported a significant difference between musicians and non-musicians on the QuickSIN task [−0.5 vs. 0.2 dB SNR Loss (estimated from Figure 1) and −0.2 (percussionists)/−0.1 (vocalists) vs. 0.25 dB SNR Loss (estimated from Figure 2A), respectively]. The overall advantage for musicians compared to non-musicians in all three of these studies ranges from 0.32 to 0.7 dB SNR Loss. In the current study the FTmus advantage over Nmus was 0.19 dB SNR Loss, while the advantage for STmus over Nmus was 0.40 dB SNR. Accordingly, the non-significant differences between FTmus, STmus and Nmus reported in the current study are consistent with previous work, and supports the idea that musicians may have a small advantage over non-musicians for understanding speech in noise.

One interesting observation from Figure 1 in Coffey et al. (2017a) is that there seems to be a nearly universal neurophysiological advantage for musicians during signal/speech in noise tasks when there is no background noise. Similarly, a recent music training study in older adults revealed that 6 months of music training could improve the ability to understand speech in loud multi-talker babble noise; however, the neurophysiological data (EEG and fMRI) revealed speech processing differences after musical training that were not impacted by the level of background noise, including when there was no background noise (Fleming et al., 2019; Zendel et al., 2019). Overall, this pattern suggests that the musician advantage for understanding speech-in-noise, may really be an advantage at understanding speech. Due to ceiling effects of behavioral speech-in-noise tests when there is no background noise or quiet background noise, differences between musicians and non-musicians only manifest behaviorally in the most difficult listening conditions. Moreover, inter-subject variability will increase as background noise level increases, making it less likely to find statistically significant differences between groups. It is therefore likely that formally trained musicians have a small advantage at processing speech sounds, which translates into a small advantage for understanding speech in background noise, and this advantage is dependent on the sample of participants. If there is a small advantage for understanding speech-in-noise in musicians, then our data suggest that this advantage occurs in both formally trained and self-taught musicians. Future studies exploring the underlying source of this putative benefit in musicians could also explore if the mechanisms that contribute to enhancements in understanding speech in noise are similar or different in STmus and FTmus.

## Conclusion

Previous work examining musicians have reported many auditory processing advantages compared to non-musicians. Some research has found that amateur, but still formally trained musicians exhibit similar auditory processing advantages over non-musicians compared to professional musicians (Oechslin et al., 2013; Rogenmoser et al., 2017). Other work has found that different specializations in music, such as style or instrument, impact the auditory processing advantages observed in formally trained musicians (Slater and Kraus, 2016; Tervaniemi et al., 2016). Here we added to this body of work by comparing formally trained musicians to self-taught musicians.

Overall, we found that musicians, regardless of training type, have advantages over non-musicians when performing tasks that were musical. Interestingly, the advantage for STmus was not as great as the advantage for FTmus compared to Nmus, for Accuracy (i.e., detecting a tonal violation) and, for the P600 evoked by an Out-of-tune note. The findings suggest that self-taught musicians exhibit advantages over non-musicians for auditory processing tasks that involve musical stimuli, but these advantages are not as great as the advantage for formally trained musicians over non-musicians. Differences between STmus, FTmus and Nmus were not found for tasks that rely on automatic stages of auditory processing (i.e., ERAN, MMN). Similarly, no differences were observed between the groups for understanding speech in noise; however, there may be a small trend toward a musician advantage when the data from the current study are considered along with other studies that compared musicians and non-musicians on the QuickSIN. These findings are critical because there is growing interest in using music-based forms of auditory rehabilitation for older adults. Results from the current study suggest that formal training may not be required to achieve some of the auditory benefits of being a musician, and that there may be limits on the effectiveness of using music training for auditory rehabilitation because differences between musicians and non-musicians were not observed universally across all the tasks in the current study.

## Data Availability Statement

The raw data supporting the conclusions of this article will be made available by the authors, without undue reservation, to any qualified researcher.

## Ethics Statement

The studies involving human participants were reviewed and approved by the Grenfell Campus Research Ethics Board (GC-REB), Grenfell Campus, Memorial University. The patients/participants provided their written informed consent to participate in this study.

## Author Contributions

BZ and EA designed the study, analyzed and interpreted the data, and wrote the manuscript. Both authors contributed to the article and approved the submitted version.

## Conflict of Interest

The authors declare that the research was conducted in the absence of any commercial or financial relationships that could be construed as a potential conflict of interest.
